# The Instantaneous Treatment of the Itch by Bergamot

**Published:** 1864-11

**Authors:** 


### THE INSTANTANEOUS TREATMENT OF THE ITCH BY BERGAMOT.

[Translated from the Journal de Medecine de Bordeaux for June, 1864.]

Dr. Manfre, the venerable clinical professor in the University of Naples, has published, in a Roman political newspaper, many articles on the rapid cure of itch. The best remedy, which he says he has thus far tried with complete success in his clinical service, is, according to M. Manfre, the oil of Bergamot, which cures *instantly*, or at most in *two minutes*, even where the eruption is general.

According to him, this remedy, is more economical, less irritating, more prompt in its insecticide effects than Helmerich's ointment or sulphur, makes the wards appropriated for patients with this disease in hospitals superfluous; for a single friction over the whole affected surface is sufficient to effect a perfect cure. The patient may return home immediately after this application, the precaution being taken of making him change his clothing, or of thoroughly purifying that which he has worn. An ounce or two of oil of bergamot is enough to complete the cure.

According to M. Manfré, the same remedy may be advantageously substituted for all those employed for the destruction of the *pediculus pubis*.

For a long time physicians have known the insecticide power of the essential oils, and there may be found in some formularies many receipts of M. Aubé for the cure of itch in two minutes. The essential oil of turpentine, mixed with essence of lemon, is the basis of the treatment recommended by this author. Before him, M. Gras had recommended the essential oil of lavender, which is quite analagous to that of bergamot, and has the additional advantage of not costing more than a quarter or half as much. —*Boston Medical and Surgical Journal.*

